# ProBiS-ligands: a web server for prediction of ligands by examination of protein binding sites

**DOI:** 10.1093/nar/gku460

**Published:** 2014-05-26

**Authors:** Janez Konc, Dušanka Janežič

**Affiliations:** 1 National Institute of Chemistry, Hajdrihova 19, 1000 Ljubljana, Slovenia; 2 University of Primorska, Faculty of Mathematics, Natural Sciences and Information Technologies, Glagoljaška 8, 6000 Koper, Slovenia

## Abstract

The ProBiS-ligands web server predicts binding of ligands to a protein structure. Starting with a protein structure or binding site, ProBiS-ligands first identifies template proteins in the Protein Data Bank that share similar binding sites. Based on the superimpositions of the query protein and the similar binding sites found, the server then transposes the ligand structures from those sites to the query protein. Such ligand prediction supports many activities, e.g. drug repurposing. The ProBiS-ligands web server, an extension of the ProBiS web server, is open and free to all users at http://probis.cmm.ki.si/ligands.

## INTRODUCTION

The problem of predicting ligands of a protein is one of the most challenging problems in biolochemistry, with profound implications for pharmaceutical chemistry and the discovery of protein function. Many approaches have been developed for protein–ligand binding prediction, the most prominent being molecular docking (1). In template-free docking, however, every new molecule must be docked *ab initio*, and information from existing similar protein–ligand complexes is not considered. The number of protein structures in the Protein Data Bank (PDB) is increasing rapidly (2) and approaches that use information from existing experimental protein–ligand complexes—an alternative to the molecular docking approach—are becoming increasingly important. In these alternative approaches, it is assumed that similar binding sites are likely to bind similar ligands and in such cases, a known ligand of one protein can be transposed to a similar binding site in another protein that was previously not known to bind this ligand. Such transposition of ligands, especially between non-homologous proteins, depends on accurate alignments of 3D patterns of amino acid functional groups in the proteins’ binding sites. Such alignments are not detectable by standard sequence or structure alignment approaches.

A recent review (3) identified several novel methods that allow transposition of ligands between protein binding sites by means of protein structure alignment (4–8). These methods can be used for drug repurposing (9–11), ligand homology modeling (12–14), template-based protein–protein docking (15–17) or protein function prediction (18–20). However, an open-access web server that can examine a database of ligands and their corresponding binding sites, automating the task of ligand transposition between similar binding sites, is not yet available.

The ProBiS algorithm (21), implemented in the ProBiS web server (22) has been described previously. It compares the query protein structure to entries in the non-redundant PDB (nr-PDB), and detects structures in this database that share similar 3D amino acid motifs with the query protein. The nr-PDB, updated weekly, currently contains more than 37 000 representative single chain protein structures in clusters with >95% sequence identity. In ProBiS, the compared proteins are represented as protein graphs, i.e. as structures of vertices and edges, where vertices correspond to functional groups of surface amino acid residues, and edges are determined by distances between vertices. A maximum clique algorithm is used for efficient comparison of these protein graphs (23). In this way, whole protein structures, in addition to pre-selected binding sites, can be compared and this enables the detection of novel similar binding sites independently of protein folding.

In this work, we describe the ProBiS-ligands web server that identifies ligands capable of binding to a query protein structure. ProBiS-ligands requires a query protein structure or a query binding site, and this is first compared to proteins in the nr-PDB using the local structural alignment algorithm ProBiS, resulting in a list of similar representative protein structures that share similar 3D amino acid environments with the query protein. Using these nr-PDB proteins as queries, all ligands are then sought in the newly prepared database, which consists of ligands from the entire PDB—proteins, nucleotides, small molecules and ions—mapped to the nr-PDB structures to which they can bind. The predicted ligands found are then transposed to the query protein by rotation and translation of their atoms’ coordinates governed by the superimposition matrices acquired from the initial superposition of the query and the nr-PDB proteins. They are then clustered according to their type and location in 3D space, and the binding amino acid residues common to the query and the source protein from which the ligand was transposed, are identified. The ProBiS-ligands web server provides an interactive environment in which users can explore the predicted protein–ligand complexes.

## THE ProBiS-LIGANDS WEB SERVER

**Figure 1.**
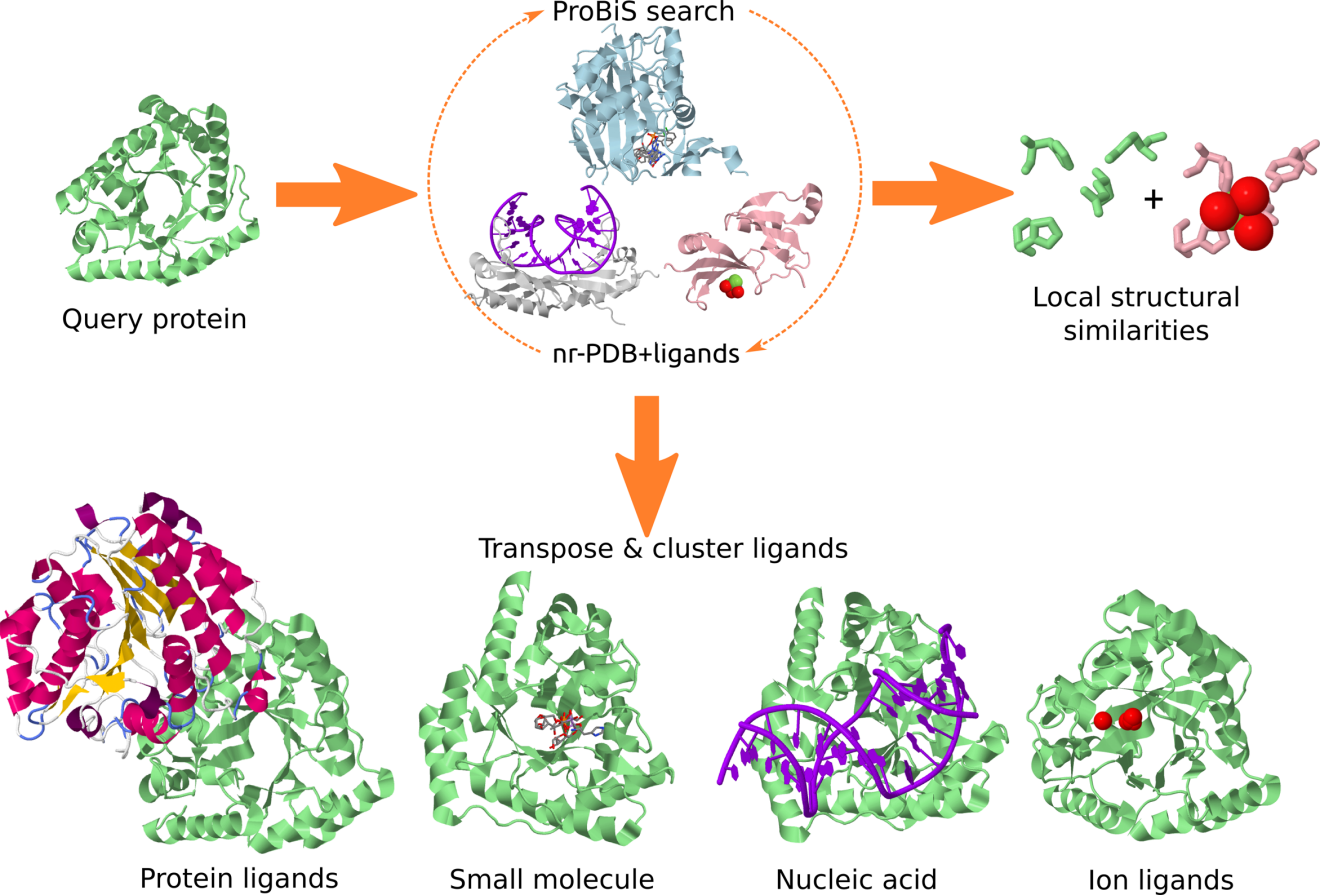
Ligand prediction by the ProBiS-ligands server, starting from a query protein structure (light green).

The input to the ProBiS-ligands server is a PDB/Chain ID or an uploaded PDB model or a selected binding site. Figure 1 is a schematic overview of the procedure followed on the ProBiS-ligands server, which includes search against the nr-PDB and then transposition of ligands to the query protein structure. In addition to the *de novo* calculation, ProBiS-ligands allows the user to see pre-calculated results instantly using the PDB ID as query through its integration with the ProBiS-Database (24).

ProBiS-ligands first identifies the template proteins with similar (*Z*-score > 1.0) patterns by comparing the query protein with all the protein chains in the nr-PDB using ProBiS algorithm (21). Second, it transposes the ligands from a ligand-to-nr-PDB mapping library to the query protein. The ligand-to-nr-PDB mapping library was prepared by dividing all proteins in the PDB into currently ∼200 000 individual protein chains. For each protein chain, all its protein–ligand complexes are generated using the symmetry rules in the corresponding PDB file, and all molecules within 4 Å of that protein chain are considered to be ligands. The biological assemblies together with the corresponding ligands are then superimposed on their representative nr-PDB chains using the ProBiS algorithm, and based on this alignment, a mapping between amino acid residues of representative and non-representative chains is established and finally, the aligned protein chains are removed. In this way, a database of ∼37 000 nr-PDB protein chains with mapped ligands was obtained from the entire PDB.

The transposition of a ligand according to this mapping library is accomplished by rotation and translation of its atoms’ coordinates according to the superimposition matrix between the query and the source, nr-PDB protein. To discard low quality alignments, the ligand is only transposed if the number of aligned residues within 4 Å of the ligand is >3 for ions, >3 for small molecules and >7 for proteins and nucleic acid ligands. The transposed ligands bound to the query protein are then clustered according to their geometric centers (separately for each ligand type) in the 3D space using a fast density clustering algorithm with a distance cutoff of 5 Å for proteins and nucleic acids, and 3 Å for small molecules and ion ligands (25). Predicted ligand clusters are listed for each ligand type and *Z*-scores are used to evaluate the predicted ligands in the query binding site. Finally, for each ligand, the invariant binding site residues, that is, residues that are <4 Å from the ligand and are a structural match between the query and the source protein, are identified.

## OUTPUT

**Figure 2.**
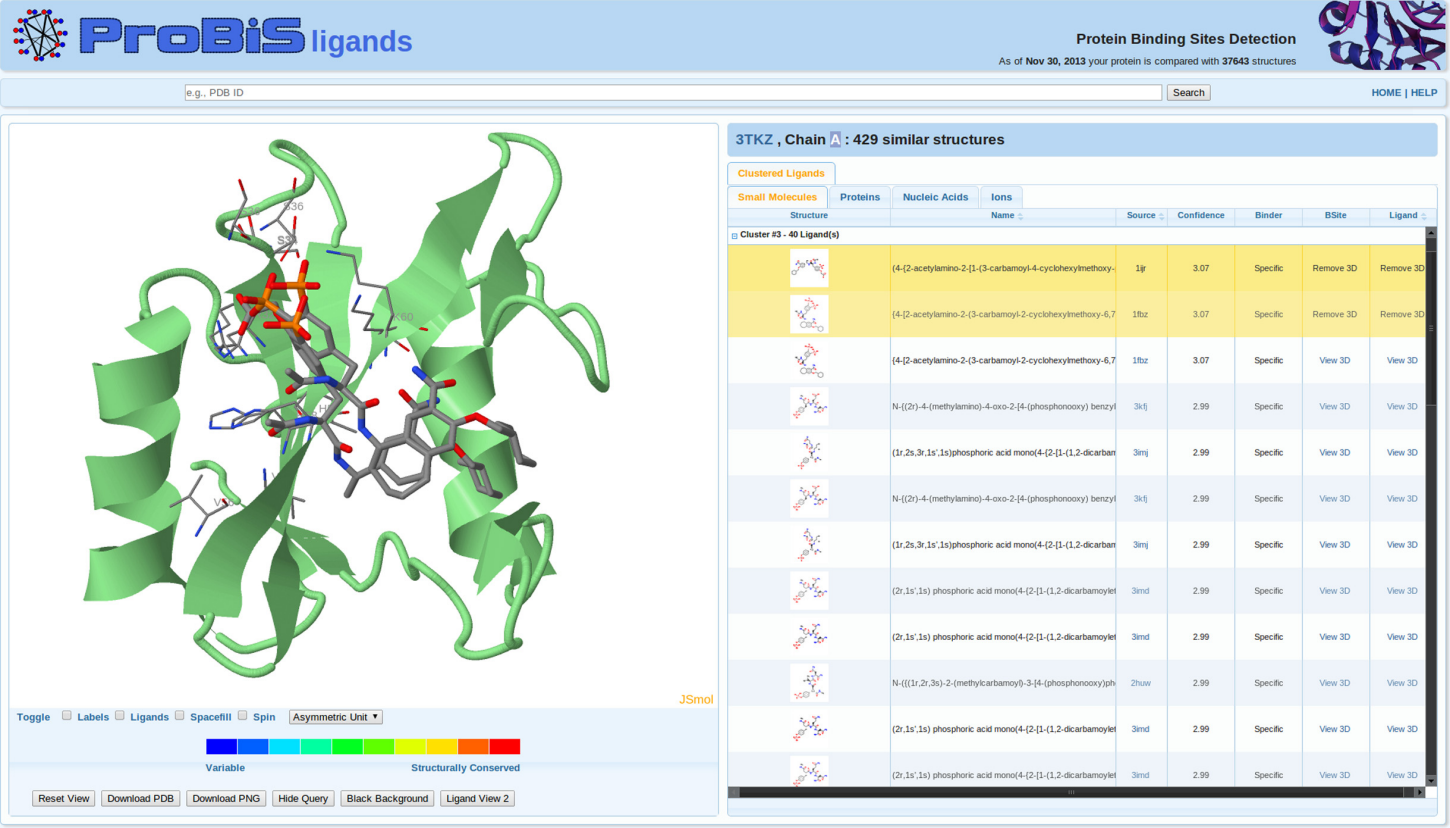
ProBiS-ligands output page. Left: query protein (green cartoon model) and two predicted ligands (CPK colored stick models). Invariant binding sites residues are thinner CPK-colored sticks. Right: table with predicted small-molecule ligands clustered according to their predicted location on the query protein and transposed from different binding sites; the two selected ligands are highlighted.

The ProBiS-ligands output page contains on the left side the *Ligand 3D Viewer* and on the right side the *Ligand Tabs* (Figure 2).

### Ligand 3D viewer

The 3D query protein (green cartoon model) and the predicted ligands (CPK colored sticks) are visualized in an integrated JSmol molecular viewer (26). This viewer uses HTML5 to display 3D molecular structures and does not require any plug-in to be installed in the browser. The invariant binding site residues from the source proteins (from which the ligands were transposed) are shown as CPK sticks. Several other options for coloring and styling the structures in the viewer are available below the viewer as buttons, e.g. *Ligand View 1*, *2* and *Conservation View*. The latter shows the query protein colored according to the degrees of structural conservation from unconserved (blue) to structurally conserved (red), as in the ProBiS web server (22). The user can choose to download the PDB file containing the structures that are currently in the viewer by using the *Download PDB* button. In addition, the *Download PNG* button downloads an image of the molecules currently in the viewer.

### Ligand tabs

On the right side of the output page, clicking on the *Small Molecules*, *Proteins*, *Nucleic Acids* or the *Ions* tab opens the corresponding interactive table, one for each ligand type; a table for small-molecule ligands is shown in Figure 2. Ligands are clustered according to their geometric centers, so that those that bind to a similar location in the query protein are in the same cluster. Clicking on the *View 3D* link in the *Ligand* column shows the predicted ligand in the *Ligand 3D viewer*, and zooms in on the ligand; the *View 3D* link in the *BSite* column shows invariant binding site residues for the corresponding ligand. The *Name* column has the names of the ligands, and *Structure* column shows a small picture of the 2D ligand structures that is enlarged when a mouse cursor is over it.

## LIGAND PREDICTION

**Figure 3.**
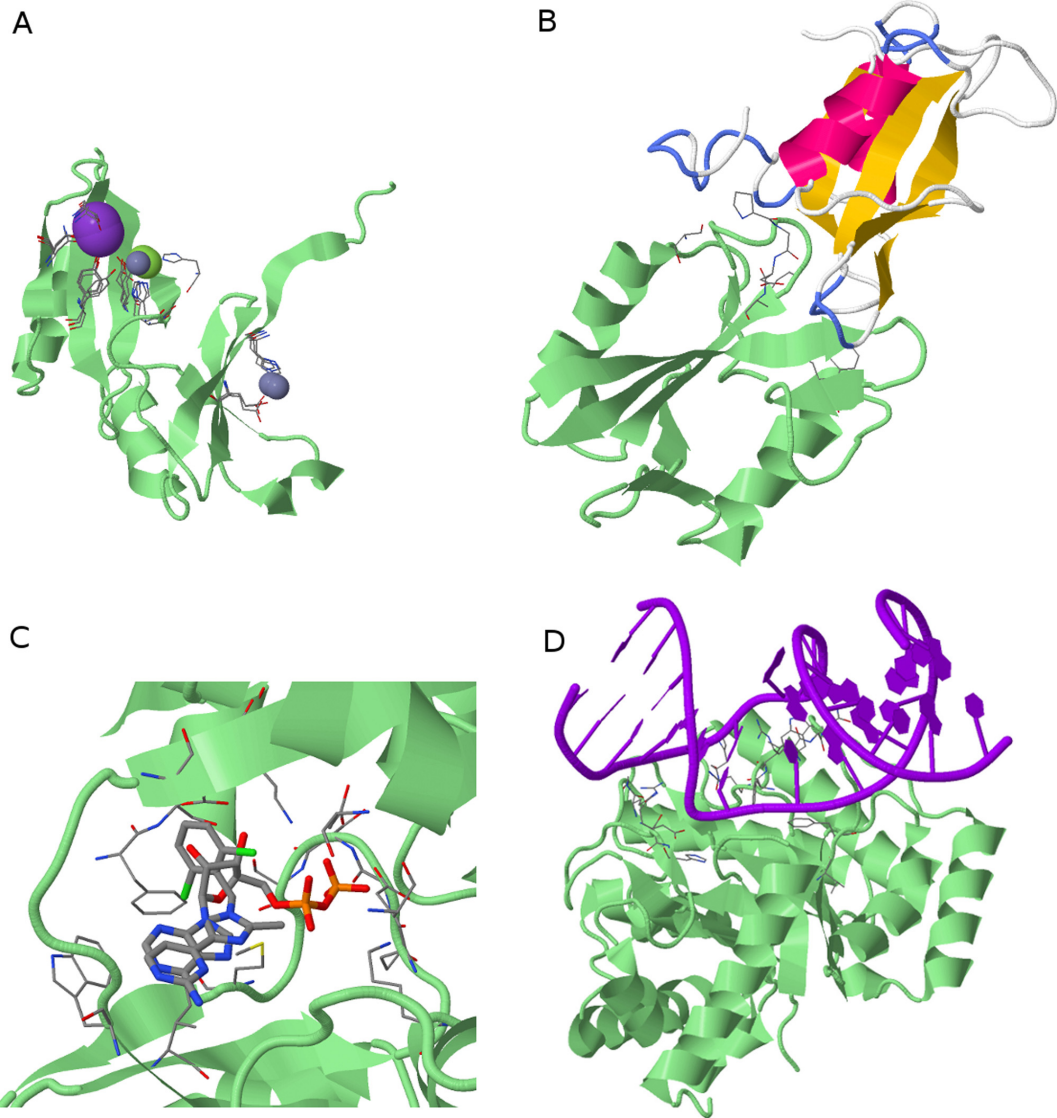
Predicted protein–ligand complexes. Query proteins are green cartoon models and invariant binding site residues are CPK-colored stick models (*Ligand View 1*). (**A**) Three predicted ion ligand clusters (ions are spheres) on *Glyoxalase family* protein (PDB ID: 2qqz). (**B**) Predicted protein ligand (yellow–pink cartoon) on *SH2 domain* protein (3tkz). (**C**) Two predicted small molecule ligands, i.e. ATP and an inhibitor of biotin carboxylase (thick CPK sticks) on *D-alanine:D-alanine* ligase (1iov). (**D**) Predicted DNA ligand on *endonuclease IV* protein (4hno).

ProBiS-ligands can be used to construct protein–ligand complexes (Figure 3) by transposition of ligands between homologous as well as between non-homologous proteins. For example, a *Glyoxalase family* protein shown in panel A has no sequence homologues in the PDB; however, ProBiS-ligands predicts three ion clusters based on the detected invariant residues in various distantly related glyoxalases. In panel B, the query protein (3tkz) is an *SH2 domain* and the predicted ligand is a protein transposed from the source PDB (1r1s) which shares ∼30% sequence identity with the query protein. This protein–protein complex is not seen in 3tkz and similar protein structures, and the possibility of its existence probably has been overlooked previously. Panel C shows a binding site in bacterial enzyme *D-alanine:D-alanine* ligase with an endogenous ATP ligand and transposed inhibitor of biotin carboxylase (<30% sequence identity). This enables one to find different fragments of inhibitors bind to the same location in a query binding site, which could be used to design new compounds as a combination of existing ligands, i.e. ligand homology modeling. Finally, in panel D, a predicted DNA ligand bound to *endonuclease IV* query protein is shown. In ProBiS-ligands, we currently do not remove steric clashes between the predicted ligands and the query proteins; we expect to address this in the future.

## PERFORMANCE OF THE WEB SERVER

We assessed the performance of the ProBiS-ligands web server on 500 protein models and their corresponding experimental structures (Table 1). This test set has been used previously for benchmarking ligand binding site prediction algorithms (27). We measured the success of ligand prediction by calculating the correspondence between the predicted ligand binding sites, i.e. query residues <4 Å from the first cluster of predicted small molecule and from the first clusters of predicted ion ligands, and the actual known binding sites for each of the 500 proteins. We evaluated the ligand binding sites prediction results using the Matthews correlation coefficient (MCC), precision and recall (for definitions see, e.g. (27)). Matthews correlation coefficient represents a score combining both the accuracy and coverage of the prediction; a coefficient of +1 represents a perfect prediction, 0 a random prediction and −1 indicates total disagreement between prediction and observation. To assess the similarity of the predicted ligands with the actual ligands, we calculated the similarity of each highest *Z*-scored predicted specific ligand from the first small molecule or ion clusters with the actual known ligands of the query proteins using an in-house developed 2D molecular graph matching algorithm. Ligand similarities, expressed as Tanimoto coefficients, were averaged over the predictions, and range between 0 and 1, where 1 is the highest similarity.

**Table 1.** Prediction of ligands by ProBiS-ligands on 500 test proteins

		Sequence identity cutoff of template proteins^a^		
		30%	20%	10%
Models	Ligand similarity^b^	0.55	0.34	0.32
	MCC	0.41	0.13	0.09
	Precision	0.42	0.17	0.14
	Recall	0.45	0.18	0.15
Experimental	Ligand similarity^b^	0.61	0.46	0.40
	MCC	0.54	0.33	0.28
	Precision	0.56	0.38	0.33
	Recall	0.57	0.36	0.31

^a^We excluded from the template libraries all protein structures with sequence identity >30%, >20% and >10% to the corresponding query proteins.

^b^Expressed with Tanimoto coefficient.

ProBiS-ligands predictions are better for experimental protein structures than for protein models, which suggests that the ProBiS algorithm is relatively sensitive to the structural accuracy of query proteins. To simulate the lack of similar templates, a situation that occurs frequently in protein structures from structural genomics projects, we consecutively excluded template proteins sharing >30%, >20% and >10% sequence identity with the query proteins from the test set. The performance dropped when similar templates were unavailable; however, for experimental query structures, reasonable predictions with MCC of 0.28 and ligand similarity of 0.40 were possible even when templates with <10% sequence identity were available; for protein models, templates with at least 20–30% sequence identity were required for similar prediction accuracy. Our results show that ProBiS-ligands predicts ligand clusters that correlate well with actual ligand binding sites even when only evolutionary unrelated templates are available. The benchmark results can be found at http://probis.cmm.ki.si/ligands/benchmark.

## CONCLUSIONS

ProBiS-ligands is a web server for prediction of ligands based on detected local structural similarities in proteins. One of the major advantages of ProBiS-ligands is that it allows transposition of ligands between protein structures irrespective of protein folding and with no prior knowledge of binding sites. This allows an established ligand, e.g. a drug, to be seen in a new perspective in which binding to other proteins, not hitherto recognized as targets, can be recognized. Repurposing of established drugs follows directly from this. We envision that the construction of accurate models of known ligands in binding sites will enable design of more specific ligands.

## ACCESSION NUMBERS

PDB IDs: 1r1s, 2qqz, 3tkz, 1iov, 4hno.
